# Family-Centered Care for LGBTQ+ Parents of Infants in the Neonatal Intensive Care Unit: An Integrative Review

**DOI:** 10.3390/children11060615

**Published:** 2024-05-21

**Authors:** Olivia Swedberg Yinger, Aubrey Jones, Keisa Fallin-Bennett, Chelsea Gibbs, Rachel H. Farr

**Affiliations:** 1 School of Music, College of Fine Arts, University of Kentucky, Lexington, KY 40506, USA; crgi236@uky.edu; 2 College of Social Work, University of Kentucky, Lexington, KY 40506, USA; aubrey.jones12@uky.edu; 3 Department of Family and Community Medicine, College of Medicine, University of Kentucky, Lexington, KY 40536, USA; keisa.bennett@uky.edu; 4 Department of Psychology, College of Arts and Sciences, University of Kentucky, Lexington, KY 40506, USA; rachel.farr@uky.edu

**Keywords:** NICU, preterm infants, neonatal, LGBTQ+ families, family-centered care, health disparities

## Abstract

Background: Having an infant in the Neonatal Intensive Care Unit (NICU) can disrupt parent well-being, the transition to parenthood, and the typical trajectories of infant and child health. For lesbian, gay, bisexual, transgender, queer, or other sexual and gender minority identity (LGBTQ+) parents, this stress may be compounded by health disparities and fear of stigma and discrimination; however, research is lacking about LGBTQ+ parents of infants in the NICU. Objectives: The purpose of this integrative review was to better understand the experiences of LGBTQ+ parents of NICU infants, with a focus on experiences of stigma and discrimination, sources of strength and resilience, and provision of family-centered care. Method: We searched EBSCOHost, ProQuest, Web of Science, and Google Scholar between 30 May 2023 and 18 September 2023 for empirical studies published in English in peer-reviewed scholarly journals in which LGBTQ+ parents shared their experiences with having infants admitted to the NICU. Results: We identified six articles that met inclusion criteria, all of which were qualitative studies that included 12–14 LGBTQ+ parents of NICU infants. Conclusions: LGBTQ+ parents in all studies reported instances of perceived stigma and discrimination while their infants were in the NICU, whereas parents in two studies mentioned strength and resilience, and parents in three studies described elements of family-centered care. There is a need for rigorous research on family-centered NICU care that includes questions about sources of strength and resilience in addition to challenges. We propose that future researchers use community engaged methods to center perspectives of LGBTQ+ parents.

## 1. Introduction

In the U.S., it is estimated that 5.5% of adults identify as lesbian, gay, bisexual, transgender, queer, or other sexual and gender minority identities (LGBTQ+) [1]. Of the 13.9 million LGBTQ+ adults [1] in the U.S., an estimated 29% are rearing children younger than 18 years of age [2]. There are many ways to become a parent (e.g., adoption, surrogacy, donor insemination, etc.), and doing so via assisted reproductive technology (ART) is increasingly common among LGBTQ+ parents [3]. Advances in reproductive technologies, adoption, and more positive attitudes toward diverse families have, in part, contributed to this increase in the use of ART [4]. In conjunction with this growth, research regarding LGBTQ+ parents and families has grown significantly. This research shows positive family outcomes, including satisfying relationships between LGBTQ+ parents and their children, engagement in positive parenting practices, and positive adjustment and development for children in LGBTQ+ families [4,5,6,7,8,9].

There is evidence that LGBTQ+ parents may be more likely to have infants admitted to the neonatal intensive care unit (NICU) than their cisgender and heterosexual (cis-het) counterparts, given their diverse pathways to parenthood. For instance, regarding foster care and adoption, same-sex partners are seven times more likely to adopt or foster children compared to different-sex partners [4]. Infants born into state custody who require foster care are more likely than other infants to be admitted to the NICU because of prenatal drug exposure or lack of prenatal care [10]. Likewise, infants are twice as likely as older children to enter foster care, most frequently due to neglect or parental substance misuse [11], which means that foster parents of infants often meet them in the NICU (or related hospital care settings). Regarding ART, LGBTQ+ parents are proportionately more likely to use forms of ART compared to their cis-het peers [12]. Infants born via ART may be more likely to require a stay in the NICU [13,14]. Lesbian women are more likely to give birth to infants with low birth weight as compared to heterosexual women [15], which is also a risk factor for time in the NICU. For all these reasons, LGBTQ+ parents—compared to cis-het parents—may be more likely to experience having an infant spend time in the NICU.

### 1.1. Family-Centered NICU Care for LGBTQ+ Families

Having an infant admitted to the NICU alters the typical transition to parenthood and can be a traumatic experience [16]. Parents of infants admitted to NICUs report high levels of stress, anxiety, uncertainty, and decreased parenting confidence [17,18,19] and may face increased risk of mental health challenges such as depression and post-traumatic stress syndrome [20,21]. These adversities have the potential to affect parent–infant bonding, infant development, and parental well-being [22], warranting a deeper evaluation of the current research related to family-centered care for LGBTQ+ parents in the NICU [23]. Family-centered care (FCC) is a philosophy of care aimed at fostering a relationship between healthcare staff and families to plan, deliver, and evaluate the healthcare provided to patients. FCC relies on four basic concepts: dignity and respect, information sharing, family participation in care, and family collaboration. When FCC is successfully implemented, families, patients, and healthcare providers benefit [24]. Researchers have begun to note the usage of FCC in health settings, and more recently, conceptualizations of FCC in the NICU have been expanded to incorporate the principles of (a) respect, (b) diversity, (c) a strengths-based approach, (d) choice, (e) flexibility, (f) information sharing, (g) support, (h) collaboration, and (i) empowerment [25,26].

Despite the growth in research regarding LGBTQ+ parenting, there is still limited knowledge regarding the needs of LGBTQ+ parents when their infants are placed in the NICU, which can lead to non-inclusive hospital policies that are at odds with LGBTQ+ parents’ rights to family-centered care, particularly as it pertains to dignity and respect [27]. For example, when required staff trainings for perinatal healthcare professionals focus on cis—and heteronormative—experiences of childbirth, infant feeding practices, and infant care, this can unintentionally exclude the experiences of LGBTQ+ parents, potentially making the transition to parenthood more stressful [27].

In the past five years, several scholars have published clinical protocols [28], case presentations [29], and insights and suggestions based on clinical wisdom and reviews of the best available literature [30,31,32,33,34] on supporting LGBTQ+ families in the NICU, during lactation, and at discharge. To provide family-centered care for LGBTQ+ parents of infants in the NICU, however, it is important to center the perspectives of LGBTQ+ parents themselves and to better understand their experiences in the NICU, particularly since family-centered care emphasizes parent empowerment and the formation of an effective therapeutic relationship with the health team [23]. Researchers have identified gaps in family-centered care for families of color and from low socioeconomic status who have had infants in the NICU [35], but the perspectives of LGBTQ+ parents of infants in the NICU regarding family-centered care have not been studied. To our knowledge, the experience of having a child in the NICU has not been explored among LGBTQ+ parent families through an integrative review. In this paper, we address this gap in the literature by presenting an integrative review of research on the experiences of LGBTQ+ families in the NICU, which we hope will provide information on the provision of family-centered care for LGBTQ+ parents. In articulating our rationale for this review, we first describe research on health disparities and experiences of stigma and discrimination among LGBTQ+ families, as well as potential sources of support and resilience for LGBTQ+ families.

### 1.2. Health Disparities, Stigma, and Discrimination among LGBTQ+ Families

Health disparities are “preventable differences in the burden of disease, injury, violence, or opportunities to achieve optimal health that are experienced by socially disadvantaged populations” [36], including LGBTQ+ people. LGBTQ+ people often present with poorer mental and physical health outcomes compared to their cisgender and heterosexual counterparts [37]. These health disparities are a result of social and systemic barriers that negatively impact LGBTQ+ individuals and lead to inequitable treatment based on sexual orientation, gender identity, or gender expression (SOGIE). Health equity is the aspirational idea that all people should have the highest possible standard of health, with special attention paid to the needs of people who are at the greatest risk of poor health based on social conditions. [38]. To achieve health equity, obstacles to health must be removed [39], and health disparities must be eliminated.

LGBTQ+ individuals in general [40], and specifically LGBTQ+ parents [41], experience stigma, discrimination, and denial of their civil and human rights based on SOGIE, which may culminate in disparate health outcomes. Stigma happens when the labeling of human differences and negative stereotyping of those differences lead to separation, status loss, and discrimination of labeled people [42]. Individuals who identify as LGBTQ+ are often stigmatized due to their group membership and their non-conformity with cis-het norms [43]. Luoma et al. [44] described and defined three forms of stigma (enacted, self, and perceived). Self-stigma refers to negative thoughts and feelings about oneself that come from identification with a stigmatized group. Perceived stigma refers to beliefs that members of a stigmatized group have about stigmatizing attitudes and actions from others in society. Enacted stigma is directly experienced social discrimination, such as interpersonal rejection or difficulty accessing healthcare, employment, or housing. Discrimination can be thought of as the unjust or prejudicial treatment of people on the grounds of their demographic identity, including sexual orientation and gender identity.

Unfortunately, stigmatizing and discriminatory experiences persist across a wide range of settings for LGBTQ+ individuals. A 2020 study found that 15% of the 1528 LGBTQ+ participants indicated they had postponed or avoided medical treatment due to fear of discrimination [45]. Accounts of NICU clinicians revealed that this fear of discrimination is not unfounded and may occur in the NICU. In a study by Sigurdson and colleagues, some NICU nurses reported discomfort with non-heteronormative families that lead to disparities in care, with 4% of clinician accounts addressing disparities in care related to sexual orientation or family status and 4% addressing disparities in care related to gender. Many nurses who participated in this study suggested that “biased attitudes and offensive language likely result in vulnerable families spending less time in the unit with their babies or engaging less with NICU clinicians because of a lack of trust and rapor [sic] with clinicians” (p. 4, [46]). The types of neglectful care, judgmental care, and systemic barriers to care that Sigurdson and colleagues revealed exist in the NICU highlight a lack of equitable family-centered care for LGBTQ+ parents. These disparities in care likely lead to avoidance or postponement of perinatal care, which could lead to negative health outcomes for infants and parents in LGBTQ+ parent families [46,47,48].

Neonatal clinicians and family advocates have identified SOGIE and family status as factors related to disparities in NICU quality of care [46]; coupled with the added discrimination facing LGBTQ+ parents, we assert that LGBTQ+ families are at a higher risk for deleterious health outcomes associated with their infant’s admittance into an NICU. LGBTQ+ parents may also draw from unique sources of support and resilience in coping with stressors [49,50], such as those related to having an infant child in the NICU and/or experiencing discrimination.

### 1.3. Sources of Support and Resilience for LGBTQ+ Families

Despite pervasive stigma, discrimination, and health disparities, LGBTQ+ people also demonstrate resilience in the context of adversity and often draw from unique sources of support. They do this through integrated positive LGBTQ+ identities [51], “chosen” family [50,52], a sense of LGBTQ+ community belonging [53], and affirming LGBTQ+ climates [54]. The theoretical and conceptual framework of queer family resilience [50,55] is apt for explaining positive adjustment and well-being that LGBTQ+ people, including parents and their children, often experience despite institutional and interpersonal stigma, discrimination, and bias. Considering such a strengths-based approach, and moving away from a damage-centered one [56], allows for acknowledgement of individual characteristics (e.g., flexibility, adaptability), behaviors (e.g., coping strategies), and relational processes (e.g., family communication, LGBTQ+ parent family socialization) that contribute to healthy functioning [50] amidst minority stress. Minority stress theory [57,58] posits that people with minoritized identities (e.g., sexual, gender) experience stress resulting from stigma and discrimination at interpersonal and institutional levels. Applying a minority stress framework without attending to positive qualities, experiences, and strengths, however, can inadvertently pigeonhole LGBTQ+ people and their families as without power, agency, resources, or hope [59]. Thus, it is important to understand the experiences of LGBTQ+ NICU parents specifically, including potential harm from discrimination but also wells of support and resilience. Generating this understanding represents one step toward decreasing barriers to family-centered, inclusive NICU care and health equity for this population. The purpose of this integrative review is to better understand the experiences of LGBTQ+ parents of NICU infants. Specifically, we addressed the following research question and sub-questions:

Based on published, peer-reviewed, empirical research, what is known about the experiences of LGBTQ+ parents when their infants are in the NICU? Specifically, to what extent and in what ways…

…do LGBTQ+ parents experience stigma and discrimination when their infants are in the NICU?

…do LGBTQ+ parents draw on sources of strength and resilience when their infants are in the NICU?

…is family-centered care discussed in the relevant literature on LGBTQ+ parents of infants in the NICU?

## 2. Materials and Methods

We engaged in the following stages of integrative review outlined by Whittemore and Knafl [60]: (1) problem identification, (2) literature search, (3) data evaluation, (4) data analysis, and (5) presentation. To promote transparency and increase methodological rigor, we also attempted to adhere to PRISMA guidelines for systematic reviews [61] when appropriate. As this review was exploratory, we did not register or publish our review protocol prior to this publication.

### 2.1. Inclusion and Exclusion Criteria

We determined the following inclusion criteria for our integrative review:

- Peer-reviewed articles in scholarly journals;
- Published in English;
- Primary empirical research (qualitative, quantitative, mixed-methods, or descriptive);
- Focused on the experiences of LGBTQ+ parents of NICU infants.

We acknowledge that only including peer-reviewed articles published in scholarly journals increases the risk of publication bias; nevertheless, we felt it was appropriate to exclude theses and dissertations to (a) add a component of quality control through the peer-review process and (b) focus on research that healthcare professionals would be most likely to access via scholarly journals. In the interest of time and because we chose to conduct an integrative review rather than a scoping review, we did not search conference proceedings or white papers. We had no exclusion criteria related to the years of publication or the country in which the study was conducted. Given the resources available to the research team, we chose to focus on articles published in English. Exclusion criteria consisted of the following:

- Articles not published in English;
- Secondary research (reviews);
- Expert opinion;
- Case studies;
- Theses or dissertations;
- Gray literature (conference proceedings, white papers);
- Research that included LGBTQ+ parents as participants but did not provide information about their experiences in the NICU;
- Research on NICU parents’ experiences that did not specify whether participants identified as LGBTQ+.

### 2.2. Search Strategy

We searched EBSCOhost, ProQuest, and Web of Science databases between 30 May 2023 and 5 June 2023 for articles with the following terms in the article text: (LGBT* OR lesbian OR gay OR bisexual OR transgender* OR transsexual OR queer OR intersex OR asexual OR pansexual OR non-binary OR “sexual and gender minority” OR SOGIE) AND (parent OR family) AND (NICU OR “neonatal intensive care unit”). We used database search filters to limit our search to peer-reviewed articles published in English. Follow-up searches between 30 August 2023 and 18 September 2023 of Google Scholar using the terms (LGBT OR lesbian OR gay OR bisexual OR transgender OR queer OR intersex OR asexual OR pansexual OR non-binary OR “sexual minority” OR “sexual and gender minority” OR SOGIE) AND NICU revealed over 1000 hits for many search terms; since there were no eligible studies apparent in the Google Scholar results after the first few pages of results, we only looked at the first eight pages of each of the Google Scholar searches.

**Figure 1:**
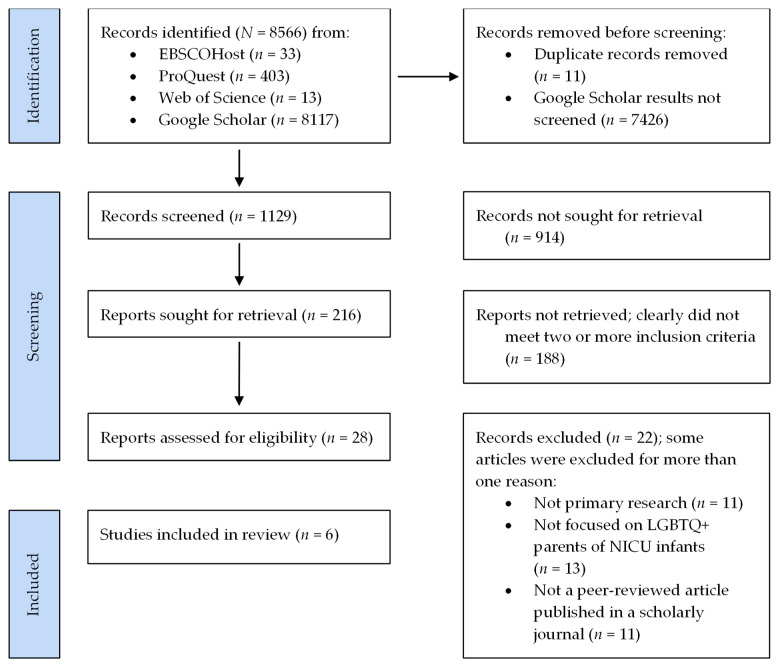
Flowchart of study identification, screening, and inclusion.

See Figure 1 for a flowchart of study identification, screening, and inclusion of articles [61]. One author (first) identified, screened, and retrieved records after consulting with a research librarian regarding the search strategy. Next, to reduce risk of bias, two of the authors (first, last) independently assessed each of the 28 records retrieved for eligibility. To manage bias in the rating process, we first created a scoring sheet with eligibility criteria for each individual record, including whether each record represented (yes/no) (a) primary research (not a review, meta-analysis, or case presentation), (b) peer-reviewed research published in a scholarly journal (not an editorial, white paper, conference proceeding, dissertation or thesis, or other non-peer-reviewed work in a newsletter or other source), and (c) included samples of LGBTQ+ identified parents of NICU infants (not other samples of parents or not focused on experiences in the NICU specifically). Individual records (*n* = 28) were then independently reviewed and rated by each of the two authors, who examined the source of each record, the type of research or content represented, and the samples included. If records were rated as yes for all three overarching criteria, they were deemed eligible for inclusion in our integrative review (if any criterion was scored as no, the record was ineligible). The two raters had 100% agreement in their ratings of inclusion criteria across the 28 records.

## 3. Results

**Figure 2:**
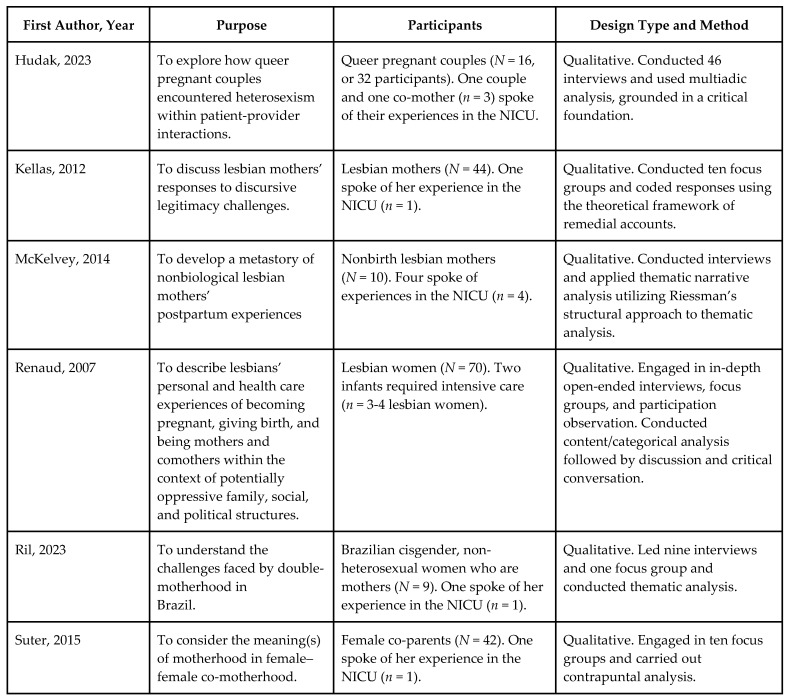
Details of included studies [62,63,64,65,66,67].

We identified six qualitative research studies, published in English in peer-reviewed scholarly journals, in which LGBTQ+ families of infants in the NICU were interviewed about their experiences. Characteristics of included studies are shown in Figure 2. Studies that initially appeared to meet inclusion criteria during the screening phase but were excluded after assessment for eligibility are shown in Supplementary Materials (Figure S1).

Although there was a total of 150 participants across these six studies, only 12 to 14 participants, all of whom identified as cisgender women, discussed having infants in the NICU. We report the range 12 to 14 participants because it appears that one group of authors shared the experiences of the same participant, Kendra, in two studies [62,63], and in one study [64], the authors reported the number of infants admitted to the NICU (two) rather than the number of parents of these infants interviewed (three or four, depending on whether they interviewed two couples or a couple and a parent, which is unclear). Although some included studies presented findings on broader experiences of LGBTQ+ parents outside of acute healthcare settings, here we solely report those findings related to LGBTQ+ parents’ experiences in the NICU, beginning with the earliest study.

### 3.1. Findings from Included Studies

Renaud [64] wrote about childbearing experiences of lesbian families. Using a critical ethnography design, she interviewed 21 English-speaking self-identified lesbian women from cities and rural communities in the Pacific Northwest, conducted focus groups with three lesbian couples, and observed an estimated 43 participants in a support group. Two babies of participants interviewed by Renaud required stays in the NICU. One couple had a positive NICU experience, whereas the other couple’s experience was less positive. Components of the positive NICU experience included the co-mother being able to see the baby immediately after delivery and having healthcare professionals encourage her to speak to the baby, acknowledging that the baby would recognize the co-mother’s voice because she had been present during the entire pregnancy. Having NICU staff treat both the birth mother and the co-mother equally well, involving them in the care of their infant immediately after birth, was another important part of this lesbian couple’s positive NICU experience. The other family whose child was admitted to the NICU reported that next-of-kin rules prevented the co-mother from visiting their infant in the NICU.

Kellas and Suter [62] studied lesbian, bisexual, and sexually fluid mothers over the course of 10 focus groups with 44 female co-parents at urban universities in two primarily rural states, using the theoretical framework of remedial accounts to discuss external family challenges. One of the challenges that emerged was related to healthcare master narratives. One non-biological mother, Kendra, whose son spent several weeks in the NICU reported that she did not get to hold her son the first day he was born, even though her partner, the birthing parent, did. When she asked why she could not hold her son, Kendra was told she “wasn’t family”. She reported that this “was probably the most negative experience” she had ever had. The healthcare provider’s challenge to Kendra’s legitimacy as a parent made this already stressful time (during which she was worried about whether her son would have brain damage and ever be able to walk or talk) even more stressful and negative. Kendra had to prove her legitimacy as a parent by demonstrating that she had Power of Attorney for her partner, at which point the doctor gave her information about her son in the NICU.

Suter and colleagues [63] later published a study on competing discourses of co-mothers based on the focus groups they described in Kellas and Suter [62]. They shared Kendra’s account of not being allowed to hold her son in the NICU as an example of how the discourse of essential motherhood defines authentic motherhood based on biology, wherein the biological mother is the only real mother.

McKelvey [65] interviewed ten nonbirth lesbian mothers from nine different states to develop a meta story of their postpartum experiences within the first year after their child’s birth. Four participants had infants who were admitted to the NICU, and all spoke of the experience as a positive one. Characteristics of NICU care that made the experience positive for nonbirth mothers were being (a) included in their babies’ care, (b) recognized as equal mothers, and (c) treated with respect. One participant spoke of the NICU experience being one that “affirmed [her] as a mother”, since the nonbirth mother was asked to accompany their baby to the NICU, make decisions, provide information, and report back to her family because the birth mother had not yet delivered the placenta. Another described a crib card that NICU nurses made that listed both mothers’ names; as a new mother, this gesture felt very special. Although parents described experiences in the NICU as positive, one participant spoke of the financial hardship that their baby’s stay in the NICU caused, exhausting the family’s financial savings, which made the cost of the second-parent adoption process more of a financial strain.

Hudak [66] wrote about heterosexism in healthcare settings, interviewing 16 queer pregnant couples from 12 different states together and separately and engaging in critical analysis to identify themes surrounding patient–provider communication and heterosexism. One couple (Sydney and Amber) reported that when their child was admitted to the NICU, Amber, the co-mother, had a stressful encounter with a security guard outside the NICU who doubted Amber’s assertion that she was the child’s mother because she had not just given birth. This interaction demonstrates how heterosexist assumptions about motherhood can make the NICU experience even more stressful for queer parents. Another co-mother, Ava, talked about how grateful she was to the nurse who showed her how to provide basic care for her son in the NICU (testing his blood sugar and temperature, changing his diaper, and feeding him), stating that this helped her feel included and like the nurse considered her a mom.

Ril et al. [67] sought to better understand the experience of double motherhood, which is the “shared experience of motherhood between two non-heterosexual cisgender women” (p. 2) by interviewing and conducting online focus groups with nine Brazilian cisgender lesbian or bisexual women who were mothers. One participant, Cloe, shared that she was not allowed to go with her daughter to the NICU because Cloe was a non-biological mother.

**Figure 3:**
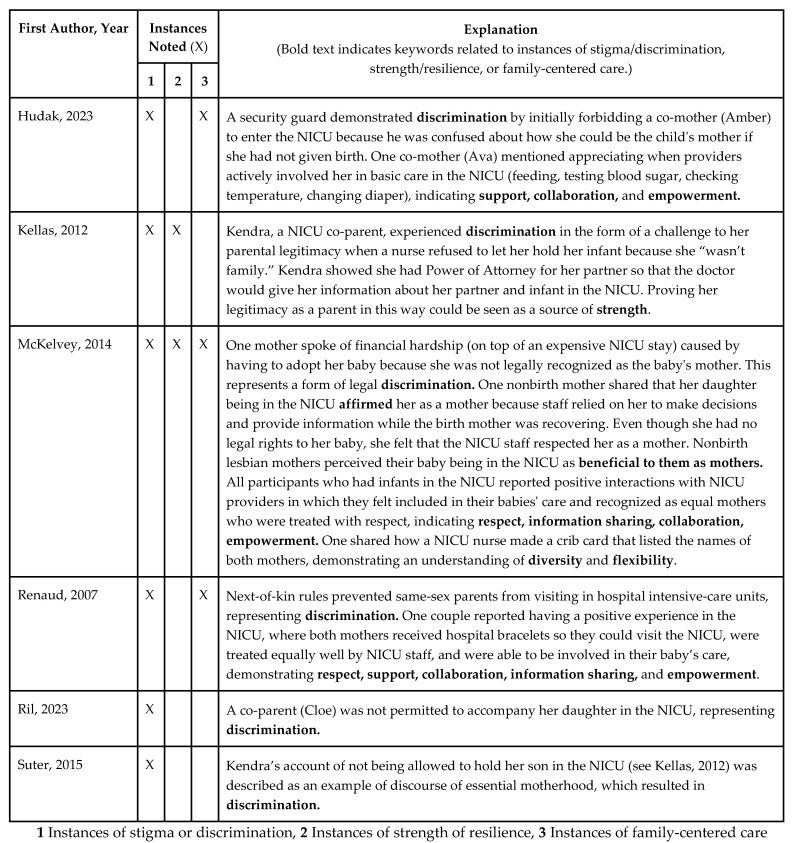
Instances of stigma/discrimination (1), strength/resilience (2), and family-centered care (3) for LGBTQ+ parents of NICU infants in included studies [62,63,64,65,66,67].

**Figure 4:**
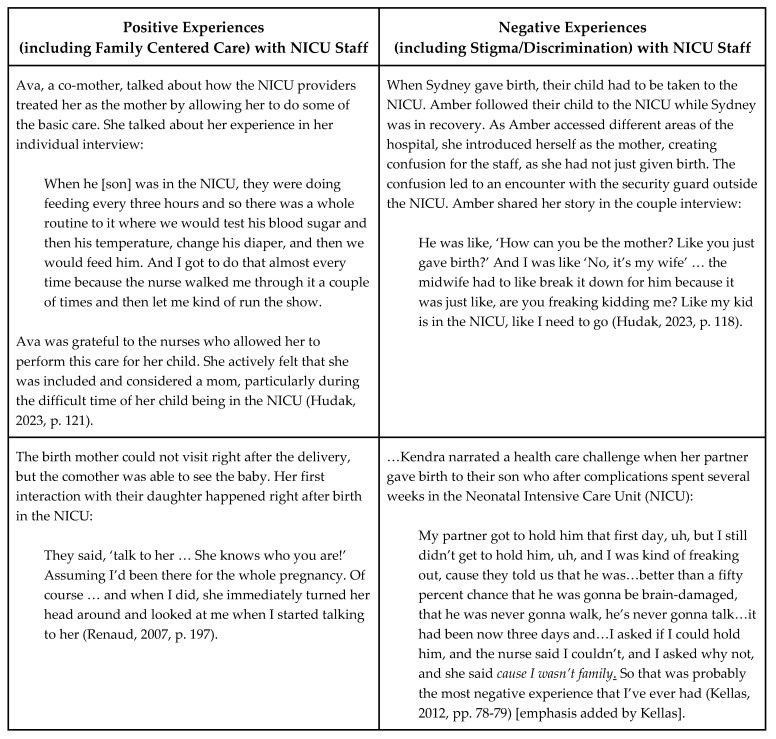
Quotes from LGBTQ+ parents who were participants in included studies about positive or negative experiences with NICU staff [62,64,66].

In the next two figures, we summarize the connection between the findings of the included studies described above and our sub-questions regarding LGBTQ+ parents’ (a) experiences of stigma and discrimination, (b) sources of strength and resilience, and (c) perceptions of family-centered care when their infants were in the NICU (see Figure 3), in addition to including quotes from parents in included studies related to positive and negative experiences with NICU staff (Figure 4).

### 3.2. Rigor of Included Studies

Because all the included studies were qualitative, we did not assess risk of bias; instead, here we report efforts authors took to ensure rigor based on criteria appropriate for qualitative research. Authors of five of the six studies reported steps they took to ensure rigor, although none specifically used the term rigor. McKelvey [65] reported that they followed Riessman’s criteria for trustworthiness, which consisted of persuasiveness, coherence, correspondence, pragmatic use, and social justice. Renaud [64] included a table outlining steps they took to ensure validity, specifically (a) prolonged engagement in the field, (b) triangulation of data sources, (c) distinguishing observations from researcher’s interpretation, (d) peer debriefing, (e) looking for negative cases, (f) thick description, (g) using non-leading questions, (h) audit trail, (i) ironic validity, (j) rhizomatic validity, (k) paralogic validity, and (l) voluptuous validity. Kellas and Suter [62] reported steps they took to establish validity of their coding scheme and to calculate unitizing reliability and intercoder reliability. To ensure the validity of their findings, Suter and colleagues [63] reported that the research team (a) maintained an analytic audit trail of data analysis and analytic decisions, (b) included data exemplars in the results section to demonstrate the link between raw data and analysis, and (c) used investigator triangulation and referential adequacy to verify the initial thematic analysis and interplay analysis. We were not able to find specific mentions of steps taken to enhance rigor in the studies by Hudak [66] and Ril and colleagues [67].

## 4. Discussion

In this study, we sought to learn about the experiences of LGBTQ+ parents with NICU infants; in particular, we examined the experiences of stigma, discrimination, resilience, and family-centered care reported in six studies that focused on NICU experiences and included LGBTQ parents. Here, we discuss the findings from the included studies in the context of our research questions and discuss connections between our findings and the existing literature.

### 4.1. Stigma and Discrimination Reported by Parents in Included Studies

We found that descriptions of discrimination (also sometimes known as enacted stigma) emerged from participants from all six studies within our review. There is extensive research showing that perceived discrimination is associated with decrements in health [68]. Previous research has identified that LGBTQ+ individuals experience dissatisfaction with healthcare settings, are less likely to seek healthcare services compared to non-LGBTQ counterparts, and experience negative communication with healthcare professionals [45,47,48].

We would be remiss if we did not discuss the effects of the U.S. Supreme Court case Obergefell v. Hodges [69] and the impact this decision has had on legal discrimination in healthcare for LGBTQ+ individuals in the United States. Obergefell v. Hodges dealt with the constitutionality of state bans on same-sex marriage. As a result of the 2015 decision, same-sex marriage was effectively legalized across the United States. This landmark case profoundly impacted the legal landscape regarding LGBTQ+ rights in the U.S. Importantly, some obstacles to health equity were removed or minimized (i.e., access to federal marriage equality for same-gender couples, access to joint parent adoptions as same-gender partners across all 50 states). One implication for LGBTQ+ parents with hospital birthing experiences (among others) is that when same-gender partners who become parents are legally married, the birth certificate of their child can reflect both partners as the parents, guaranteeing non-birthing parents their parental rights. Four studies included in our review were conducted in the U.S. prior to this 2015 case [62,63,64,65]. Parents in these studies mentioned experiences that we would (likely) expect no longer occur in the U.S. as a result of Obergefell v. Hodges; namely, co-mothers in these studies were not considered the legal parents of their infants because they could not legally marry the child’s birthing mother and were denied certain parental rights in the NICU (e.g., holding their infant, caring for their infant). However, even though the study by Hudak [66] was published more than five years after the Obergefell v. Hodges decision, Amber, a parent interviewed by Hudak, reported experiencing discrimination from an NICU security guard because her child had two mothers.

### 4.2. Sources of Strength and Resilience Reported by Parents in Included Studies

Parents in two of the included studies [62,65] alluded to sources of strength or resilience during their infants’ NICU experiences. In the study by Kellas and Suter [62], one co-mother, Kendra, showed she had Power of Attorney for her partner so that the doctor would give her information about her partner and infant in the NICU; proving her legitimacy as a parent in this way could be seen as a source of strength via knowledge and resources. In McKelvey’s [65] article, one nonbirth mother shared that her daughter being in the NICU affirmed her as a mother because staff relied on her to make decisions and provide information while the birth mother was recovering. Even though she had no legal rights to her baby, the nonbirth mother felt that the NICU staff respected her as a mother. Nonbirth lesbian mothers in McKelvey’s study perceived their babies being in the NICU as beneficial to them as mothers because it helped legitimize their roles as parents [65]. These experiences highlight the importance of LGBTQ+ parents having choice, agency, self-efficacy, decision-making power, and the trust of staff in the context of having an infant in the NICU, all of which constitute sources of strength and resilience. In addition, these findings align with the broader literature about the lengths that LGBTQ+ parents frequently employ to ensure legal and practical security for their families [50].

Although we were able to infer sources of strength from the included studies, none of the authors of the included studies specifically named sources of strength or resilience as such. This could be because authors of five of the included studies designed their interview questions to elicit information about challenges experienced by LGBTQ+ parents, namely heterosexism [66]; discursive legitimacy challenges [62,63]; being mothers and co-mothers within the context of potentially oppressive family, social, and political structures [64]; and challenges faced by double-motherhood [67]. By contrast, McKelvey [65] simply asked participants to tell the story of their postpartum experiences, without explicit focus on challenges faced. However, none of the authors of included studies specifically asked participants about sources of strength and resilience when their infants were in the NICU.

### 4.3. Examples of Family-Centered NICU Care in Included Studies

We also sought to understand what the current literature could reveal about the use of family-centered care for LGBTQ+ parents in the NICU. As noted in the background, family-centered care is a philosophy of care aimed at fostering a relationship between healthcare staff and families to plan, deliver, and evaluate the healthcare provided to patients, and it relies on four main principles (dignity and respect, information sharing, family participation, and collaboration). In only one study [65] in our review did the authors explicitly discuss family-centered care. The authors posited the importance of engaging in family-centered care with LGBTQ parents. However, in other studies, LGBTQ+ parents of infants in the NICU mentioned positive interactions with healthcare professionals in which they experienced care in line with the principles of FCC. For example, some participants in the included studies reported having positive experiences in the NICU when hospital staff (a) affirmed their roles as parents by showing them how to care for their children [65,66] and (b) treated both mothers equally well, including both mothers’ names on the infant’s crib card [65] or acknowledging that the baby would recognize the non-birthing mother’s voice as well as the birthing mother’s voice [64].

Being able to see the baby immediately after delivery [64] and helping to make decisions about their infants’ care [65] also made participants feel respected and affirmed as parents during their infant’s NICU stay. As such, in contrast to experiences of discrimination, we suggest that NICU and hospital personnel engaging in family-centered care can contribute to building resilience among LGBTQ+ parents with a baby in the NICU. Practitioners can emphasize unique strengths among LGBTQ+ parents and cultivate their connection to various support resources, which may be LGBTQ+-specific. When LGBTQ+ parents feel supported, and have access to necessary resources, these dynamics promote the positive health and development of their NICU infants. These processes are not necessarily unique to LGBTQ+ parents, yet LGBTQ+ parents may be able to draw from unique sources of support, such as from having a positive, integrated LGBTQ+ identity; connections and sense of belongingness to LGBTQ+ community; and chosen family [50].

### 4.4. Limitations of This Integrative Review

Our integrative review should be considered within the context of its limitations. First, the relevant literature that met inclusion criteria was limited, and all studies were qualitative. The few articles on the topic point to a need to focus future research on the LGBTQ+ parent experience in the NICU, including larger and broader samples of LGBTQ+ parents. Second, our search was restricted by the authors’ language limitations. The authors are native English speakers and do not fluently speak other languages; thus, our search was limited to articles written in English. We acknowledge that only including articles in English may have limited our results in terms of geographic and cultural contexts. Our findings must also be considered in the context of the participants captured in each individual study. Participants from all six articles identified as cisgender women, thus limiting the understanding of trans or non-binary individuals and gay men who may have adopted a child placed in the NICU. Lastly, we did not include “gray literature” in our search, thus limiting the potential studies conducted through dissertations, thesis work, or from non-academic institutions and organizations. Despite these limitations, our study contributes to the body of research literature, and our limitations point to important considerations for future research.

### 4.5. Suggestions for Future Research

Given that so few studies (*n* = 6) have directly addressed the experiences of LGBTQ+ parents who have an infant in the NICU, and so few participants were represented in these six studies (*n* = 12–14), as revealed by our integrative review, more research is needed on this topic. We recommend further qualitative, descriptive, and mixed-methods research to develop a larger body of evidence related to LGBTQ+ parents of NICU infants. Conducting research with the population through community-based participatory research methods would be ideal, as participants can work with researchers to inform the direction of the research and identify the needs of the community (i.e., research with participants, not on them). Furthermore, in light of past harm caused to the LGBTQ+ community by certain researchers [5], there is an ethical imperative for researchers to engage in community-engaged research practices that ensures respect and recognition of members of the LGBTQ+ community by centering their perspectives and including them throughout the research process. Research related to healthcare professionals who work with this population is greatly needed, as is research on perceived stigma among LGBTQ+ parents of NICU infants. Finally, the literature regarding LGBTQ+ individuals and their families more broadly needs to center on a strengths-based perspective. As it stands, much of the research is deficit-focused, despite the strength and resilience exhibited by the LGBTQ+ population [50].

### 4.6. Suggestions for Healthcare Professionals and LGBTQ+ Parents

Here, we highlight recommendations that parents in the included studies made for parents and healthcare professionals to center the perspectives of LGBTQ+ parents. Participants in Kellas and Suter’s [62] study offered suggestions to other female coparents, including (a) be yourself and be a model for others, (b) manage your emotions, (c) surround your family with positive people, and (d) focus on the kids. Renaud [64] reported that participants had negative experiences with the healthcare system in general, which has implications for NICU providers, including (a) intake forms that were not inclusive of lesbian partners, (b) staff not understanding issues important to lesbian families, and (c) a lack of recognition of the partner if the birth mother or infant experienced an emergency, death, or illness. This information has implications for parents, who may unfortunately still be faced with navigating these challenges, and for healthcare providers, who can be proactive in removing these obstacles for LGBTQ+ parents in the NICU to promote health equity.

Parents interviewed by Hudak [66] offered the following advice for healthcare professionals: (a) include the co-parent, (b) actively involve both parents in infant care, (c) screen co-parents for depression, (d) treat partners as equals, (e) pay attention to language and assumptions, (f) ask rather than assume, and (g) make notes for future visits so that parents do not have to constantly explain. Other suggestions from parents interviewed by Hudak included (a) asking LGBTQ+ parents open-ended questions to better understand what is important to them; (b) using parent’s pronouns; (c) acknowledging any mistakes, apologizing, and moving on; and (d) advocating for queer parents. The interaction that Amber and Sydney, mothers interviewed by Hudak, had with the security guard highlights the need for training in the provision of LGBTQ+-affirming care to involve all staff in a healthcare system who may engage with parents, not just physicians and nurses.

## 5. Conclusions

The purpose of this integrative review was to examine the current literature regarding the experiences of LGBTQ+ parents of NICU infants. Of primary importance, we sought to understand experiences of stigma, discrimination, strength, resilience, and family-centered care. Our search yielded six articles that met the inclusion criteria. Participants from all six studies discussed experiences that can be categorized as stigma or discrimination, whereas parents in two studies discussed experiences that were indicative of strength and resilience. Although participants in three studies described experiences consistent with principles of family-centered care, only one author specifically noted family-centered care. Our findings highlight the need for more methodologically diverse, community-engaged research on the perspectives of LGBTQ+ parents in the NICU setting.

## Data Availability

The raw data supporting the conclusions of this article will be made available by the authors on request.

## Supplementary Materials

The following supporting information can be downloaded at https://www.mdpi.com/article/10.3390/children11060615/s1, Figure S1: Studies that initially appeared to meet inclusion criteria during the screening phase but were excluded after assessment for eligibility. References [70,71,72,73,74,75,76,77,78,79,80,81,82,83,84] are cited in Supplementary Materials.

## References

[1] Flores A.R., Conron K.J. (2023). Adult LGBT Population in the United States.

[2] Williams Institute (2019). LGBT Demographic Data Interactive.

[3] Patterson C.J., Farr R.H., Goldberg A.E. (2021). LGBTQ+ Parents and Their Children.

[4] Goldberg S.K., Conron K.J. (2018). How Many Same-Sex Couples in the U.S. Are Raising Children?. https://williamsinstitute.law.ucla.edu/publications/same-sex-parents-us/.

[5] Goldberg A.E., Allen K.R. (2020). LGBTQ-Parent Families: Innovations in Research and Implications for Practice.

[6] Patterson C.J. (2000). Family Relationships of Lesbians and Gay Men. J. Marriage Fam..

[7] Golombok S. (2015). Modern Families: Parents and Children in New Family Forms.

[8] Carone N., Lingiardi V., Chirumbolo A., Baiocco R. (2018). Italian Gay Father Families Formed by Surrogacy: Parenting, Stigmatization, and Children’s Psychological Adjustment. Dev. Psychol..

[9] Farr R.H. (2017). Does Parental Sexual Orientation Matter? A Longitudinal Follow-Up of Adoptive Families with School-Age Children. Dev. Psychol..

[10] Marcellus L. (2010). Supporting Resilience in Foster Families: A Model for Program Design that Supports Recruitment, Retention, and Satisfaction of Foster Families who Care for Infants with Prenatal Substance Exposure. Child Welf..

[11] Williams S.C., Sepulveda K. (2019). Infants and Toddlers Are More likely than Older Children to Enter Foster Care Because of Neglect and Parental Drug Abuse. https://www.childtrends.org/blog/infants-and-toddlers-are-more-likely-than-older-children-to-enter-foster-care-because-of-neglect-and-parental-drug-abuse.

[12] Raja N.S., Russell C.B., Moravek M.B. (2022). Assisted Reproductive Technology: Considerations for the Nonheterosexual Population and Single Parents. Fertil. Steril..

[13] Ibi K., Takahashi N. (2023). Assisted Reproductive Technology and Neonatal Intensive Care Unit: A Retrospective Observational Study from a Single Center. J. Obstet. Gynaecol. Res..

[14] Scala M., Berg J., Keszler M., Abubakar K. (2019). Premature Infants Conceived with Assisted Reproductive Technology: An Analysis of Infant Morbidity, Compared with Infants Conceived Naturally. Am. J. Perinat..

[15] Everett B.G., Kominiarek M.A., Mollborn S., Adkins D.E., Hughes T.L. (2019). Sexual Orientation Disparities in Pregnancy and Infant Outcomes. Matern Child Health J..

[16] Hynan M.T., Hall S.L. (2015). Psychosocial Program Standards for NICU Parents. J. Perinatol..

[17] Ghorbani M., Dolatian M., Shams J., Alavi-Majd H., Tavokolian S. (2014). Factors Associated with Posttraumatic Stress Disorder and its Coping Styles in Parents of Preterm and Full-term Infants. Glob. J. Health Sci..

[18] Janvier A., Lantos J., Aschner J., Barrington K., Batton B., Batton D., Berg S.F., Carter B., Campbell D., Cohn F. (2016). Stronger and More Vulnerable: A Balanced View of the Impacts of the NICU Experience on Parents. Pediatrics.

[19] Lundqvist P., Hellström-Westas L., Hallström I. (2014). Reorganizing Life: A Qualitative Study of Fathers’ Lived Experience in the 3 Years Subsequent to the Very Preterm Birth of their Child. J. Pediatr. Nurs..

[20] Law K.H., Dimmock J.J., Guelfi K.J., Nguyen T., Gucciardi D., Jackson B. (2018). Stress, Depressive Symptoms, and Maternal Self-efficacy in First-time Mothers: Modelling and Predicting Change Across the First Six Months of Motherhood. Appl. Psychol. Health Well Being.

[21] Malin K.J., Johnson T.S., McAndrew S., Westerdahl J., Leuthner J., Lagatta J. (2020). Infant Illness Severity and Perinatal Post-traumatic Stress Disorder after Discharge from the Neonatal Intensive Care Unit. Early Hum. Dev..

[22] Vance A.J., Knafl K., Brandon D.H. (2020). Patterns of Parenting Confidence Among Infants with Medical Complexity: A Mixed-methods Analysis. Adv. Neonatal. Care.

[23] Gómez-Cantarino S., García-Valdivieso I., Moncunill-Martínez E., Yáñez-Araque B., Gurrutxaga M.I.U. (2020). Developing a Family-centered Care Model in the Neonatal Intensive Care Unit (NICU): A New Vision to Manage Healthcare. Int. J. Environ. Res. Public Health.

[24] Griffin T. (2006). Family-centered Care in the NICU. J. Perinat. Neonatal. Nurs..

[25] Franck L.S., O’Brien K. (2019). The Evolution of Family-centered Care: From Supporting Parent-delivered Interventions to a Model of Family Integrated Care. Birth Defects Res..

[26] Gooding L.F., Yinger O.S., Standley J. (2023). Family-centered NICU care: Supporting Parents and Families. Evidence-Based Music Therapy for Premature Infants.

[27] Jackson J.R., Moreno L., Camen M., Dadiz R. (2022). What are LGBTQI+ Parental Experiences of Healthcare Support and Decision-making Regarding Infant Feeding Options? A Grounded Theory Study. J. Perinatol..

[28] Ferri R.L., Rosen-Carole C.B., Jackson J., Carreno-Rijo E., Greenberg K.B. (2020). ABM Clinical Protocol #33: Lactation Care for Lesbian, Gay, Bisexual, Transgender, Queer, Questioning, Plus Patients. Breastfeed Med..

[29] Paul K.J., Murosko D., Smith V.C., Montoya-Williams D., Parga-Belinkie J. (2023). Defining Gender in Infant Care. Neoreviews.

[30] Kyle B., Dowling D.A. (2022). Inclusivity and Respect: Beyond Personal Pronouns. Adv. Neonatal. Care.

[31] Logan R. (2020). Gay Fatherhood in the NICU: Supporting the “Gayby” Boom. Adv. Neonatal. Care.

[32] Smith V.C., Love K., Goyer E. (2022). NICU Discharge Preparation and Transition Planning: Guidelines and Recommendations. J. Perinatol..

[33] Smith V.C., Litt J.S. (2018). Insights and Suggestions to Support Lesbian, Gay, Bisexual, Transgender, and Queer/Questioning (LGBTQ) Parents in the NICU. Neonatol. Today.

[34] Smith V.C., Litt J.S., Wylie M.F. (2019). Further Insights and Suggestions to Support Lesbian, Gay, Bisexual, Transgender, and Queer/Questioning (LGBTQ)-headed Families in the NICU. Neonatol. Today.

[35] Sigurdson K., Profit J., Dhurjati R., Morton C., Scala M., Vernon L., Randolph A., Phan J.T., Franck L.S. (2020). Former NICU Families Describe Gaps in Family-centered Care. Qual. Health Res..

[36] Centers for Disease Control and Prevention (2023). Health Disparities. https://www.cdc.gov/healthyyouth/disparities/index.htm.

[37] Medina-Martínez J., Saus-Ortega C., Sánchez-Lorente M.M., Sosa-Palanca E.M., García-Martínez P., Mármol-López M.I. (2021). Health Inequities in LGBT People and Nursing Interventions to Reduce Them: A Systematic Review. In. J. Environ. Res. Public Health.

[38] Braveman P. (2014). What are Health Disparities and Health Equity? We Need to be Clear. Public Health Rep..

[39] Braveman P., Arkin E., Orleans T., Proctor D., Acker J., Plough A. (2018). What is Health Equity?. Behav. Sci. Policy.

[40] U.S. Department of Health and Human Services (2021). Healthy People 2030: Goal: Improve the Health, Safety, and Well-Being of Lesbian, Gay, Bisexual, and Transgender People. https://health.gov/healthypeople/objectives-and-data/browse-objectives/lgbt.

[41] Assink M., Rothblum E.D., Wilson B.D.M., Gartrell N., Bos H.M.W. (2022). Mental Health of Lesbian, Bisexual, and Other-identified Parents and Non-parents from a Population-based Study. J. Homosex..

[42] Link B.G., Phelan J.C. (2001). Conceptualizing Stigma. Annu. Rev. Sociol..

[43] Worthen M.G.F. (2020). Queers, Bis, and Straight Lies: An Intersectional Examination of LGBTQ Stigma.

[44] Luoma J.B., Twohig M.P., Waltz T., Hayes S.C., Roget N., Padilla M., Fisher G. (2007). An Investigation of Stigma in Individuals Receiving Treatment for Substance Abuse. Addict. Behav..

[45] Gruberg S., Mahowald L., Halpin J. (2020). The State of the LGBTQ Community in 2020: A National Public Opinion Study. https://www.americanprogress.org/article/state-lgbtq-community-2020/.

[46] Sigurdson K., Morton C., Mitchell B., Profit J. (2018). Disparities in NICU Quality of Care: A Qualitative Study of Clinician Accounts. J. Perinatol..

[47] Goldberg A.E. (2022). LGBTQ Family Building: A Guide for Prospective Parents.

[48] Ross L.E., Goldberg A.E., Wenzel A. (2016). Perinatal Experiences of Lesbian, Gay, Bisexual, and Transgender People. The Oxford Handbook of Perinatal Psychology.

[49] Farr R.H., Tornello S.L., Nolan M., Gore S. (2023). The Transition to Parenthood and Early Child Development in Families with LGBTQ+ Parents. Contemporary Issues in Perinatal Education: Knowledge for Practice.

[50] Farr R.H., Tornello S.L., Rostosky S.S. (2022). How do LGBTQ+ Parents Raise Well-adjusted, Resilient, and Thriving Children?. Curr. Dir. Psychol. Sci..

[51] Siegel M., Legler M., Neziraj F., Goldberg A.E., Zemp M. (2022). Minority Stress and Positive Identity Aspects in Members of LGBTQ+ Parent Families: Literature Review and a Study Protocol for a Mixed-methods Evidence Synthesis. Children.

[52] Weston K. (1997). Families We Choose: Lesbians, Gays, Kinship.

[53] Lin Y.-J., Israel T. (2012). Development and Validation of a Psychological Sense of LGBT Community Scale. J. Community Psychol..

[54] National Academies of Science, Engineering, and Medicine (2020). Consensus Study Report: Understanding the Well-Being of LGBTQI+ Populations.

[55] Prendergast S., MacPhee D. (2018). Family Resilience Amid Stigma and Discrimination: A Conceptual Model for Families Headed by Same-sex Parents. Fam. Relat..

[56] Tuck E. (2009). Suspending Damage: A Letter to Communities. Harv. Educ. Rev..

[57] Brooks V.R. (1981). Minority Stress and Lesbian Women.

[58] Meyer I.H. (2003). Prejudice, Social Stress, and Mental Health in Lesbian, Gay, and Bisexual Populations: Conceptual Issues and Research Evidence. Psychol. Bull..

[59] Levitt H.M., Kehoe K.A., Hand A.B. (2023). Beyond Minority Stress: Toward a Multidimensional Psychology of Trans/nonbinary Gender. Curr. Opin. Psychol..

[60] Whittemore R., Knafl K. (2005). The Integrative Review: Updated Methodology. J. Adv. Nurs..

[61] Page M.J., McKenzie J.E., Bossuyt P.M., Boutron I., Hoffman T.C., Mulrow C.D., Shamseer L., Tetzlaff J.M., Akl E.A., Brennan S.E. (2021). The PRISMA 2020 Statement: An Updated Guideline for Reporting Systematic Reviews. Br. Med. J..

[62] Kellas J.K., Suter E.A. (2012). Accounting for Lesbian-headed Families: Lesbian Mothers’ Responses to Discursive Challenges. Pap. Commun. Stud..

[63] Suter E.A., Seurer L.M., Webb S., Grewe B., Kellas J.K.K. (2015). Motherhood as Contested Ideological Terrain: Essentialist and Queer Discourses of Motherhood at Play in Female-female Co-mothers’ Talk. Commun. Monogr..

[64] Renaud M.T. (2007). We are Mothers Too: Childbearing Experience of Lesbian Families. J. Obstet. Gynecol. Neonatal. Nurs..

[65] McKelvey M.M. (2014). The Other Mother: A Narrative Analysis of the Postpartum Experiences of Nonbirth Lesbian Mothers. ANS Adv. Nurs. Sci..

[66] Hudak N. (2023). “Who’s the Mom?”: Heterosexism in Patient-provider Interactions of Queer Pregnant Couples. Health Commun..

[67] Ril S.Y., Mello M.M.C., Moretti-Pires R.O. (2023). Queer Kinship: Experiences of Double-motherhood in Brazil. Sexualities.

[68] Pascoe E.A., Richman L.S. (2009). Perceived Discrimination and Health: A Meta-analytic Review. Psychol. Bull..

[69] (2015). Obergefell v. Hodges, 576 U.S. 644. https://supreme.justia.com/cases/federal/us/576/644/.

[70] Bree C. (2003). Lesbian Mothers: Queer Families- The Experience of Planned Pregnancy. Master’s Thesis.

[71] Burrow S., Goldberg L., Searle J., Aston M. (2018). Vulnerability, Harm, and Compromised Ethics Revealed by the Experiences of Queer Birthing Women in Rural Healthcare. J. Bioeth. Inq..

[72] Danna M. (2017). Queering the Birthing Body: Coming Out Fatigue and the Medical Gaze. Master’s Thesis.

[73] Dawson S.J., Leonhardt N.D., Impett E.A., Rosen N.O. (2021). Associations Between Postpartum Depressive Symptoms and Couples’ Sexual Function and Sexual Distress Trajectories Across the Transition to Parenthood. Ann. Behav. Med..

[74] Heyes C.J., Thachuk A. (2015). Queering Know-how: Clinical Skill Acquisition as Ethical Practice. J. Bioeth. Inq..

[75] Hudak N. (2021). Queer Healthcare Communication. Oxford Research Encyclopedia of Communication.

[76] Johnson A., Baldino J., Peavey M.C., Bressler L.H. (2023). Live Birth Following IVF Pregnancy in a Transgender Man of Advanced Paternal Age with a History of Prolonged Testosterone Use. Fertil. Steril..

[77] Knight M., Acosta C., Brocklehurst P., Cheshire A., Fitzpatrick K., Hinton L., Jokinen M., Kemp B., Kurinczuk J.J., Lewis G. (2016). Beyond Maternal Death: Improving the Quality of Maternal Care through National Studies of ‘Near-miss’ Maternal Morbidity. Programme Grants Appl. Res..

[78] Leonhardt N.D., Rosen N.O., Dawson S.J., Kim J.J., Johnson M.D., Impett E.A. (2022). Relationship Satisfaction and Commitment in the Transition to Parenthood: A Couple-centered Approach. J. Marriage Fam..

[79] Lorenz W.P. (2020). Dynamic Norms’ Influence on Relationship Quality during the Transition to Parenthood. Ph.D. Thesis.

[80] Norris M., Borneskog C. (2022). The Cisnormative Blindspot Explained: Healthcare Experiences of Trans Men and Non-binary Persons and the Accessibility to Inclusive Sexual & Reproductive Healthcare, an Integrative Review. Sex. Reprod. Health.

[81] Reimann J.G. (2022). Re-Riting Ritual for Reproductive Loss: An Inclusive Support Resource for Catholics Experiencing Reproductive Loss. Master’s Thesis.

[82] Rowe D.D. (2016). Please Don’t Use the Restraints: Forgetting, Failure, and Childbirth. Qual. Inq..

[83] Tarasoff L.A., Lunsky Y., Welsh K., Proulx L., Havercamp S.M., Parish S.L., Brown H.K. (2023). Unmet Needs, Limited Access: A Qualitative Study of Postpartum Health Care Experiences of People with Disabilities. J. Adv. Nurs..

[84] Upchurch J.K. (2022). Understanding Autonomy and Positionality in Obstetric Care Outcomes for Queer Individuals. Honor’s Thesis.

