# Supplementary material for: Family-Centered Care for LGBTQ+ Parents of Infants in the Neonatal Intensive Care Unit: An Integrative Review
Source: Children (Basel). 2024 May 21;11(6):615. doi: 10.3390/children11060615 (PMC11201882; doi:10.3390/children11060615)
Supplement: Supplementary file 1 [file children-11-00615-s001.zip › children-2997850-supplementary.pdf]

Supplementary Materials

First Author, Year	Information About Article and Reason for Exclusion
Bree, 2003	Qualitative study (radical hermeneutics) in which lesbian mothers were interviewed about their pregnancies. One participant's child went to the NICU after birth, but the study was not focused on LGBTQ+ parents of NICU infants. Master's thesis; not a peer-reviewed article published in a scholarly journal.
Burrow, 2018	Qualitative study (feminist phenomenology) in which queer women in rural Nova Scotia were interviewed about their birthing experiences. Not focused on LGBTQ+ parents of NICU infants.
Danna, 2017	Qualitative study in which queer birthing parents in the Pacific Northwest were interviewed about their birthing experiences. Not focused on LGBTQ+ parents of NICU infants. Master's thesis; not a peer-reviewed article published in a scholarly journal.
Dawson, 2021	Longitudinal dyadic study in which mothers and partners were surveyed about sexual function/distress and postpartum depressive symptoms from mid pregnancy until 12-months postpartum. Not focused on LGBTQ+ parents of NICU infants.
Ferri, 2020	Clinical protocol on lactation care for LGBTQ+ patients, including those with infants in the NICU. Not primary research.
Heyes, 2015	Qualitative study of queer women patients and their primary health care providers in Nova Scotia. Not focused on LGBTQ+ parents of NICU infants.
Hudak, 2021	Overview of queer healthcare communication, with recommendations for healthcare providers. Not focused on LGBTQ+ parents of NICU infants. Entry in Oxford Research Encyclopedia; not primary research published in a peer-reviewed scholarly journal.
Johnson, 2023	Case study of a transgender man's IVF pregnancy, which resulted in a NICU stay for his twin infants. Abstract from a conference presentation; not a peer-reviewed article published in a scholarly journal.
Knight, 2016	Mixed methods study of severe complications of pregnancy in which birthing women and their partners (one of whom was a lesbian) were interviewed. Not focused on LGBTQ+ parents of NICU infants. Grant report; not peer-reviewed article published in a scholarly journal.
Kyle, 2022	Editorial on using inclusive, respectful language when referring to LGBTQ+ parents in the NICU. Not peer-reviewed or primary research.
Leonhardt, 2022	Longitudinal study of relationship satisfaction/commitment through 12 months postpartum for couples (some of whom were lesbian, gay, bisexual, pansexual, asexual, or trans). Not focused on LGBTQ+ parents of NICU infants.
Logan, 2020	Literature review with recommendations on supporting gay fathers in the NICU. Not primary research.
Lorenz, 2020	Randomized controlled trial evaluating an intervention to improve relationship quality during transition to parenthood; some parents were homosexual, bisexual, or pansexual, but the study was not focused on LGBTQ+ parents of NICU infants. Not a peer-reviewed article published in a scholarly journal.
Norris, 2022	Integrative review on accessibility of inclusive sexual reproductive healthcare for trans men and non-binary people. Although the author mentioned the need for NICUs to be more inclusive, the article is not focused on LGBTQ+ parents of NICU infants.

Paul, 2023	Case study presenting relevant literature with expert commentary on challenges faced by transgender individuals during pregnancy and postpartum healthcare experiences. Not primary research.
Reimann, 2022	Inclusive support resource for Catholics experiencing reproductive loss. The author mentioned the need for resources for queer Catholics and provided spiritual care in the NICU. Not focused on LGBTQ+ parents of NICU infants. Not a peer-reviewed article published in a scholarly journal.
Rowe, 2016	Autoethnographic account of the author's negative birth experience that resulted in her daughter staying in the NICU for a week. Although the author referenced queer theory and described herself as being in a "queered heterosexual relationship, the article was not focused on LGBTQ+ parents of NICU infants.
Smith, 2018	Insights and suggestions on supporting LGBTQ parents in the NICU. Not primary research. Not published in a scholarly journal.
Smith, 2019	Further insights and suggestions on supporting LGBTQ-headed families in the NICU. Not primary research. Not published in a scholarly journal.
Smith, 2022	Consensus statement; Interdisciplinary Guidelines and Recommendations for NICU Discharge Preparation and Transition Planning, including discharge considerations specifically for LGBTQIA+ headed families. Not primary research.
Tarasoff, 2023	Qualitative study where birthing people with disabilities were interviewed about postpartum care. Some parents were trans, non-binary, lesbian, bisexual, queer, or asexual and had NICU infants. Not focused on LGBTQ+ parents of NICU infants.
Upchurch, 2022	Case study in which a cisgender lesbian woman from Eastern Tennessee was interviewed about her experience receiving obstetric care. Her child had an extended stay in the NICU. Master's thesis; not a peer-reviewed article published in a scholarly journal.

Figure S1. Studies that initially appeared to meet inclusion criteria during the screening phase but were excluded after assessment for eligibility [28-34,70-84].
